# Modeling the pre-metastatic niche of gastric cancer peritoneal metastasis under spatiotemporal resolution and investigating EVs-mediated immune suppression

**DOI:** 10.3389/fimmu.2025.1734244

**Published:** 2026-01-09

**Authors:** Xiang Zhang, Cheng Zhang, Zheng Zhang, Xuan Zhang, Kai Wang

**Affiliations:** 1 School of Anesthesiology, Wannan Medical College, Wuhu, Anhui, China; 2 School of Clinical Medicine, Wannan Medical College, Wuhu, Anhui, China; 3 School of Clinical Medicine, Bengbu Medical University, Bengbu, Anhui, China; 4 Department of General Surgery, The Affiliated Hospital of Xuzhou Medical University, Xuzhou, Jiangsu, China

**Keywords:** extracellular vesicles, gastric cancer, immune suppression, organoid modeling, peritoneal metastasis, pre-metastatic niche

## Abstract

Gastric cancer peritoneal metastasis (GCPM) is the leading cause of death in patients with advanced gastric cancer. The presence of ascites creates favorable conditions for the formation of the pre-metastatic niche (PMN), thereby providing a conducive environment for cancer cell metastasis. However, the mechanisms behind the formation of the pre-metastatic niche (PMN) are still not fully understood. This review examines the dynamic changes in the PMN of gastric cancer using organoid models combined with high spatiotemporal resolution and looks into the role of extracellular vesicles (EVs) in mediating immune suppression. It gives an overview of the latest advances in organoid modeling technologies, clarifies the biological characteristics of EVs, and discusses their functions in immune regulation. Furthermore, this review also highlights current challenges in this field, proposes future research directions, and identifies potential therapeutic targets. Bringing these insights together is intended to deepen understanding of gastric cancer metastasis and support the development of more effective therapeutic strategies.

## 1 Introduction

Gastric cancer (GC) is the sixth-most common cancer and third-leading cause of cancer-related death worldwide, as a globally prevalent malignant tumor with complex pathophysiological mechanisms (1). Importantly, peritoneal metastasis serves as a major factor contributing to poor prognosis in patients with advanced gastric cancer. It often eliminates the possibility of curative surgery and is linked to high mortality rates (2).

In the context of gastric cancer peritoneal metastasis, ascites can create necessary conditions for the formation of the pre-metastatic niche (PMN), thereby providing critical environmental support for the colonization and metastasis of cancer cells (3). Extracellular vesicles (EVs) are key mediators in PMN formation, as they not only precisely target the peritoneum through organ-specific tropism but also establish an immunosuppressive microenvironment. This function removes barriers for PMN colonization in the peritoneum and builds a communication bridge between tumor cells and distant metastatic sites. In-depth exploration of the mechanisms by which EVs regulate PMN helps clarify the rules governing the expansion and invasion of malignant tumors, and lays a foundation for the development of cancer detection methods and precision therapies (4). Traditional 2D cell culture models have significant limitations, as they cannot recapitulate the complex tumor microenvironment. In contrast, patient-derived organoids (PDOs) are 3D models with high physiological relevance. They can accurately replicate the structural and cellular characteristics of primary gastric tumors. As such, PDOs serve as a core *in vitro* platform for cancer research, effectively compensating for the shortcomings of traditional models (5). Spatiotemporal resolution technologies further enable breakthroughs in GCPM mechanism research. These technologies not only reveal the heterogeneous cellular composition of the PMN microenvironment and clarify the spatial distribution of key signaling pathways, but also enable real-time tracking of dynamic processes (e.g., EV migration and PMN formation) in living organisms (6, 7). This makes previously unobservable regulatory mechanisms accessible, significantly deepening the understanding of the pathological processes underlying GCPM.

Based on the aforementioned mechanisms and technical support, potential therapeutic strategies for GCPM have become increasingly clear, including targeting EV-related pathways to block PMN formation, modulating immune checkpoints to reverse immunosuppression, and using PDOs for personalized drug screening to guide clinical treatment decisions (8, 9). This review also identifies key challenges in current research. For example, PDOs still have limitations in recapitulating complex physiological structures such as blood vessels, and high-sensitivity detection of EVs faces technical barriers. Future research should focus on integrating multiple models and technologies, and accelerating the translation of basic research findings into clinical applications.

## 2 Clinical and molecular characteristics of gastric cancer peritoneal metastasis

### 2.1 Core risk factors associated with gastric cancer onset

Gastric cancer, characterized by its complex pathogenesis involving a multitude of risk factors, is a significant global health concern (10). Helicobacter pylori (H. pylori), the core risk factor, was isolated from gastric mucosal samples by Warren and Marshall in 1982, earning them the 2005 Nobel Prize and overturning the long-held “sterile stomach” dogma (11). Infecting approximately 50% of the global population, it spreads primarily via fecal-oral, gastro-oral, and oral-oral routes (12, 13). The novel pathogenic mechanism has recently been identified that H. pylori and infected host epithelial cells secrete extracellular vesicles (EVs) carrying CagA, VacA, adhesins, and immunomodulatory molecules. These EVs can facilitate transcellular delivery to reshape the host immune microenvironment, drive chronic inflammation, and promote malignant transformation, emerging as a key mediator directly contributing to gastric carcinogenesis (14).

**Figure 1:**
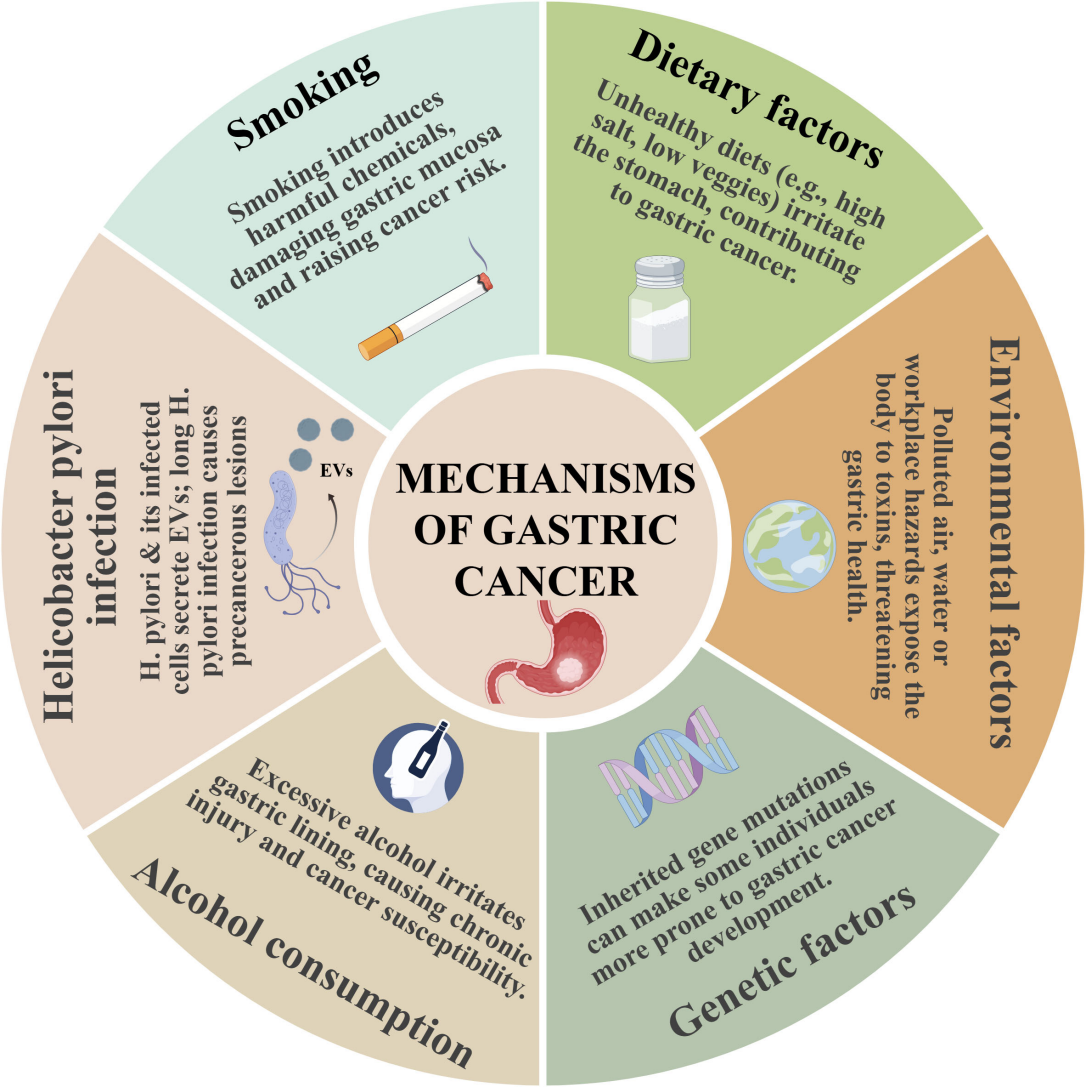
Schematic diagram of the mechanisms of gastric cancer, showing the roles of smoking, dietary factors, environmental factors, genetic factors, alcohol consumption, and *Helicobacter pylori* infection in the development of gastric cancer.

Other risk factors include heavy alcohol consumption (>4 drinks/day), smoking (with a dose-dependent risk), high-salt or pickled food intake, nitrite/nitrate exposure, and metal dust contact. However, adequate consumption of fruits, vegetables, and antioxidants may reduce the risk. Genetically, the inactivation of tumor suppressor genes (e.g., p53) is critical in gastric cancer development, and adenovirus-mediated p53 delivery has demonstrated therapeutic potential. Collectively, these pathogenic factors synergistically contribute to the onset and progression of gastric cancer (15–19) (**Figure 1**).

### 2.2 Epidemiology and prognosis of peritoneal metastasis in gastric cancer

Peritoneal metastasis is a significant complication in advanced gastric cancer, affecting over 50% of patients at this stage and contributing to a dismal prognosis, with median survival often reported to be less than six months (20, 21). Epidemiological data shows that approximately 20–30% of gastric cancer patients have peritoneal metastases at initial diagnosis, and this complication accounts for a striking 70% of deaths in patients with advanced gastric cancer (22, 23). The clinical implications of peritoneal metastasis are significant, as it not only indicates late-stage disease but also complicates treatment strategies and frequently reduces their effectiveness. Peritoneal metastasis is associated with several histopathological features, including a higher incidence of signet ring cell carcinoma—a subtype recognized for its aggressive nature and poorer outcomes than other gastric cancer types (24). Furthermore, factors like tumor grade and primary tumor location strongly influence the risk of peritoneal dissemination, and antral tumors are particularly prone to this metastatic pattern.

Notably, molecular markers have emerged as critical components in the prognosis of gastric cancer with peritoneal metastasis. For instance, the expression of CLDN18.2 and HER2 has been linked to peritoneal dissemination, providing potential targets for therapeutic intervention. CLDN18.2, a tight junction protein, plays a role in the metastatic process, while HER2 overexpression is associated with aggressive disease and poor survival outcomes. The clinical value of these markers resides in their capacity to guide treatment decisions, particularly as immunotherapies are now increasingly integrated into gastric cancer treatment algorithms (25, 26).

### 2.3 Pathophysiological mechanisms of peritoneal metastasis

The pathophysiology of peritoneal metastasis is complex and multifaceted, and it is frequently explained by the “seed and soil” hypothesis. This hypothesis postulates that tumor cells (the “seeds”) detach from the primary tumor, disseminate, and successfully colonize the peritoneal cavity (the “soil”) under favorable conditions. The peritoneal environment offers a unique niche supporting the survival and proliferation of metastatic cells. Tumor cells can shed from the primary gastric tumor and enter the peritoneal cavity, where they need to get past multiple barriers before establishing metastatic colonies. These include the mesothelial layer lining the peritoneum, which serves as both a barrier and a facilitator for tumor cell invasion. The interaction between tumor cells and mesothelial cells is critical. Mesothelial cells secrete growth factors and cytokines that support tumor cell survival and migration, in turn boosting the metastatic process (27, 28). Furthermore, the composition of peritoneal fluid, which can be altered by tumor cells, plays a critical role in shaping the tumor microenvironment. Malignant ascites fosters a supportive niche that promotes tumor cell proliferation and facilitates immune evasion, thereby complicating therapeutic efforts (29).

In addition to the “seed and soil” hypothesis, peritoneal mesothelial cells play a crucial role in peritoneal metastasis. These cells not only act as a physical barrier but also have complex interactions with cancer cells—interactions that can either inhibit or promote metastasis. In response to tumor-derived signals, mesothelial cells can undergo phenotypic changes such as mesothelial-to-mesenchymal transition (MMT), a process that enhances their migratory and invasive potential. This transition is marked by the loss of epithelial markers and the gain of mesenchymal features, letting mesothelial cells help form a fibrotic stroma that supports tumor growth (29). Also, the inflammatory response triggered by tumor cells can further alter how mesothelial cells behave, leading to an immunosuppressive microenvironment that helps tumor progression. This dynamic interaction between tumor cells and mesothelial cells highlights the importance of the peritoneal microenvironment in the pathophysiology of peritoneal metastasis, and it points to potential therapeutic targets for intervention (30).

Overall, in gastric cancer peritoneal metastasis, the key previously discussed pathological mechanisms collectively lay a critical foundation for PMN formation and EVs functions. Collectively, these mechanisms establish the structural and functional basis of the PMN by transforming the peritoneal microenvironment to support tumor colonization, disrupting mesothelial barriers, and establishing communication networks that sustain the survival of metastatic cells. This foundation enables EVs which act as regulators in later metastasis to function. Specifically, EVs leverage prior microenvironmental conditions to maintain PMN immunosuppression via miRNAs, promote angiogenesis via cytokines, and drive metastatic spread. Elucidating the interactions between these mechanisms, PMN biogenesis, and EV regulation thus provides a framework for targeted therapies, which in turn improves outcomes for patients with this refractory gastric cancer (31).

## 3 Formation and function of pre-metastatic niche

### 3.1 Spatiotemporal dynamics of PMN

The pre-metastatic niche (PMN) undergoes a sophisticated spatiotemporal evolution. Tumor-derived factors act as the central regulatory hub, orchestrating microenvironmental remodeling and immune cell recruitment across distinct stages and ultimately paving the way for successful tumor metastasis (32).

At the initiation of PMN formation, tumor cells secrete a cocktail of signaling molecules including tumor-derived growth factors (TDSF), pro-angiogenic factors such as vascular endothelial growth factor (VEGF), pro-inflammatory cytokines like interleukin-6 (IL-6), and extracellular vesicles (EVs) as key signal carriers (33, 34). This molecular cascade triggers dual spatial modifications: on one hand, it induces initial degradation and structural rearrangement of the extracellular matrix (ECM) at distant organ sites, creating preliminary spatial niches for subsequent cellular infiltration; on the other hand, inflammatory signals activate tissue-resident immune cells (e.g., macrophages) and stromal cells (e.g., fibroblasts) in target organs, reprogramming their functional phenotypes to establish the primitive “soil” for PMN colonization, thereby completing the spatial priming of the pre-metastatic niche (35). Moreover, gastric cancer-derived EVs (GC-EVs) display specific tropism toward peritoneal-targeted metastasis, with this specificity primarily mediated by integrin αvβ3 on their surface. This integrin facilitates the directional crosstalk between GC-EVs and the peritoneal microenvironment, which in turn offers key functional support for gastric cancer peritoneal metastasis (36).

During the mid-development stage of PMN, the spatial remodeling process enters an intensified phase characterized by significant ECM reorganization. Fibronectin deposition increases markedly, and the expression of matrix proteins such as tenascin C is upregulated, driving the fibrotic transformation of the ECM structure (37). Concurrently, sustained action of pro-angiogenic factors enhances local vascular permeability, which not only accelerates the spatial deposition and remodeling efficiency of ECM components but also promotes directional recruitment of myeloid-derived suppressor cells (MDSCs) to the target organ microenvironment, laying the cellular foundation for the formation of immunosuppressive spatial domains within the PMN (38). During gastric cancer (GC) peritoneal metastasis, the spatiotemporal dynamics of myeloid-derived suppressor cells (MDSCs) are synergistically regulated by three factors secreted by primary GC cells: VEGF, TGF-β, and CXCL family chemokines. These chemokines mediate the directional recruitment of MDSCs to the pre-metastatic niche (PMN) by establishing a chemokine gradient. These regulatory processes collectively support PMN formation and lay the foundation for GC peritoneal metastasis (39, 40).Additionally, macrophages are also the major sources of CXCL1 and CXCL5 in the gastric cancer microenvironment, and promote migration of gastric cancer cells through activating a CXCR2/STAT3 feed-forward loop. Gastric cancer cells secrete TNF-α to induce release of CXCL1 and CXCL5 from macrophages. Inhibiting CXCR2 pathway of gastric cancer cells can suppress migration and metastasis of gastric cancer *in vitro* and *in vivo*. Additionally, macrophages serve as major sources of CXCL1 and CXCL5 in the gastric cancer microenvironment, and promote the migration of gastric cancer cells by activating a CXCR2/STAT3 feed-forward loop. Gastric cancer cells secrete TNF-α to induce macrophages to release CXCL1 and CXCL5. Inhibiting the CXCR2 pathway in gastric cancer cells suppresses the migration and metastasis of gastric cancer *in vitro* and *in vivo (*41).

In the late maturation stage of the PMN, the secretion of local inflammatory and chemotactic signals (e.g., chemokines CXCL1, CXCL3, CXCL5, and CXCL8) peaks, driving the continuous infiltration of multiple immune cell populations into the niche. These cells include hematopoietic progenitor cells, immature myeloid cells, neutrophils, tumor-associated macrophages (TAMs), regulatory T cells (Tregs), dysfunctional dendritic cells (DCs), and natural killer (NK) cells (42, 43). These recruited immune cells engage in complex intercellular crosstalk with PMN-resident cells (such as activated fibroblasts and mesothelial cells) through cytokine exchange and surface molecule interactions, forming stable inflammatory amplification circuits and immunosuppressive spatial networks. At this stage, the ECM undergoes continuous modification to exhibit high fibrosis and heterogeneity (44, 45). With the enhancement of immune cell recruitment, the persistence of inflammatory status, and the solidification of immunosuppressive spatial domains, the PMN finally provides structural support for the adhesion and extravasation of circulating tumor cells (CTCs), and supports their survival and proliferation within the metastatic spatial niche through nutrient supply and signal regulation, completing the spatial colonization of metastatic lesions (46).

In summary, the spatiotemporal dynamics of the PMN in gastric cancer are closely linked to the secretion of tumor-derived factors—factors that recruit and activate immune cells. The interaction between these factors and the immune microenvironment is key to understanding the mechanisms behind peritoneal metastasis. By clarifying these dynamics, researchers can identify potential therapeutic targets targeted at disrupting PMN formation and enhancing anti-tumor immunity, which will ultimately help improve treatment outcomes for patients with gastric cancer (47, 48).

### 3.2 Immunosuppressive mechanisms in PMN

The expansion and function of regulatory T cells (Tregs) are crucial to the immunosuppressive mechanisms associated with polymorphonuclear MDSCs in various cancer contexts, including gastric cancer. Tregs are a specialized subset of CD4+ T cells characterized by their expression of the transcription factor FoxP3, which is essential for their development and function. In the tumor microenvironment, Tregs can be expanded by various factors, including cytokines like IL-10 and TGF-β that are often secreted by MDSCs themselves (49). This expansion results in more Tregs being present in the microenvironment, and these cells inhibit the activation and proliferation of effector T cells—ultimately creating an immunosuppressive environment that supports tumor progression. The interaction between MDSCs and Tregs works in both directions. On one hand, MDSCs can promote Treg expansion; on the other hand, Tregs can further boost the immunosuppressive activity of MDSCs by secreting immunosuppressive cytokines. This forms a feedback loop that sustains tumor immune evasion (50). Moreover, the presence of Tregs has been correlated with poor prognosis in cancer patients, as their accumulation can lead to diminished anti-tumor immune responses and increased tumor burden.

M2 macrophage polarization is also a key driver of tumor progression. Its core role is to enable tumors to evade immune surveillance by establishing an immunosuppressive tumor microenvironment, while providing essential microenvironmental support for tumor growth and metastasis. It is revealed that the role of M2 macrophages is primarily regulated by SERPINE1, which binds to lipoprotein receptor-related protein 1 (LRP1) in a paracrine manner to induce M2 macrophage polarization. Polarized M2 macrophages subsequently facilitate the formation of the TME and further suppress the infiltration and functional activity of CD8+ T cells within the TME, ultimately promoting gastric cancer progression (51).

Furthermore, in the gastric cancer peritoneal metastasis model, miR-17–92 cluster carried by EVs exerts a significant mediating effect on the formation of the PMN in gastric cancer. Its core mechanism lies in the targeted regulation of SRCIN1, followed by the subsequent modulation of macrophage function via the SRCIN1-SRC axis, ultimately providing crucial support for the establishment of the PMN (52).

Cytokines like IL-10 and IL-6 are key to regulating immune responses within the pre-metastatic niche (PMN), and they contribute to forming the immunosuppressive environment typical of cancer progression. IL-10 is widely known for its immunosuppressive properties as it inhibits the activation and function of various immune cells, such as T cells and dendritic cells, to support tumor survival and growth (52). For MDSCs, IL-10 is one of their secreted cytokines. It further reduces effector T cell activity while enhancing Treg function, and these effects collectively foster an immunosuppressive milieu. On the other hand, IL-6 is a pro-inflammatory cytokine. Though initially thought to promote immune responses, it is now known to have dual roles in cancer. Within the PMN, elevated IL-6 levels trigger activation of the STAT3 signaling pathway. This activation is linked to the expansion of MDSCs and Tregs, and this relationship thus exacerbates immune suppression (53). By disrupting the regulatory effects of IL-10 and IL-6, it may be possible to enhance anti-tumor immunity and improve the efficacy of existing cancer therapies.

### 3.3 The critical role of PMN modeling in gastric cancer research

Gastric Cancer metastasis is not only associated with the malignant progression of the primary tumor but also involves alterations in the PMN. Constructing models of this is crucial for predicting gastric cancer progression, detecting metastasis at an early stage, and formulating personalized treatment strategies (54).

Before metastasis occurs, the tumor microenvironment undergoes gradual evolution. Cancer cells promote the metastatic process by regulating surrounding cells and their secreted signals (55). Establishing models that recapitulate the gastric cancer PMN enables researchers to investigate cell-cell crosstalk, immune evasion mechanisms, and the effects of microenvironmental components on metastatic behavior. Such models not only support early diagnosis but also facilitate the personalized therapies targeting gastric cancer metastasis (56).

Organoids are a novel 3D cell culture technology that can accurately simulate the tumor microenvironment *in vitro (*57). In gastric cancer research, organoid models can effectively reproduce the interactions between cancer cells and surrounding tissues, which helps analyze key molecular changes in the PMN (58). With organoids, researchers can predict early events of gastric cancer metastasis and provide a powerful platform for screening new anti-metastatic drugs(50). In addition to organoids, 3D cell culture models, animal models, and microfluidic chips are also important tools for comprehensively evaluating cancer metastasis mechanisms and developing effective therapeutic strategies (59, 60).

### 3.4 Microenvironmental regulation and EVs’ role in peritoneal metastasis and dissemination of gastric cancer

In the stepwise dissemination of gastric cancer peritoneal metastasis, supported by synergistic actions of multiple factors in the peritoneal microenvironment, EVs act as key carriers of intercellular signals (61). They are widely distributed via ascites and humoral circulation, playing a central regulatory role in this process (62). At the initial metastatic stage, pro-metastatic factors secreted by primary tumor cells initiate epithelial-mesenchymal transition for detachment, while tumor-derived EVs enter the humoral circulation and reach the peritoneum via blood flow. Some EVs directly integrate into ascites, carrying pro-metastatic factors to pre-modify the peritoneal microenvironment and enhance mesothelial cell adhesion to tumor cells through phenotypic induction, laying the foundation for colonization (63).​For example, EVs secreted by ST3G5-high expressing cancer cells (ST3G5 high -cExos) are rich in HIF1α. After being taken up by macrophages and accumulating in peritoneal milky spots, these EVs induce pro-inflammatory factors and metabolic changes in macrophages, upregulate CCL5 via STAT3 activation, promote mesothelial-mesenchymal transition of peritoneal mesothelial cells and immunosuppression, and ultimately accelerate the peritoneal metastasis (64).

**Figure 2:**
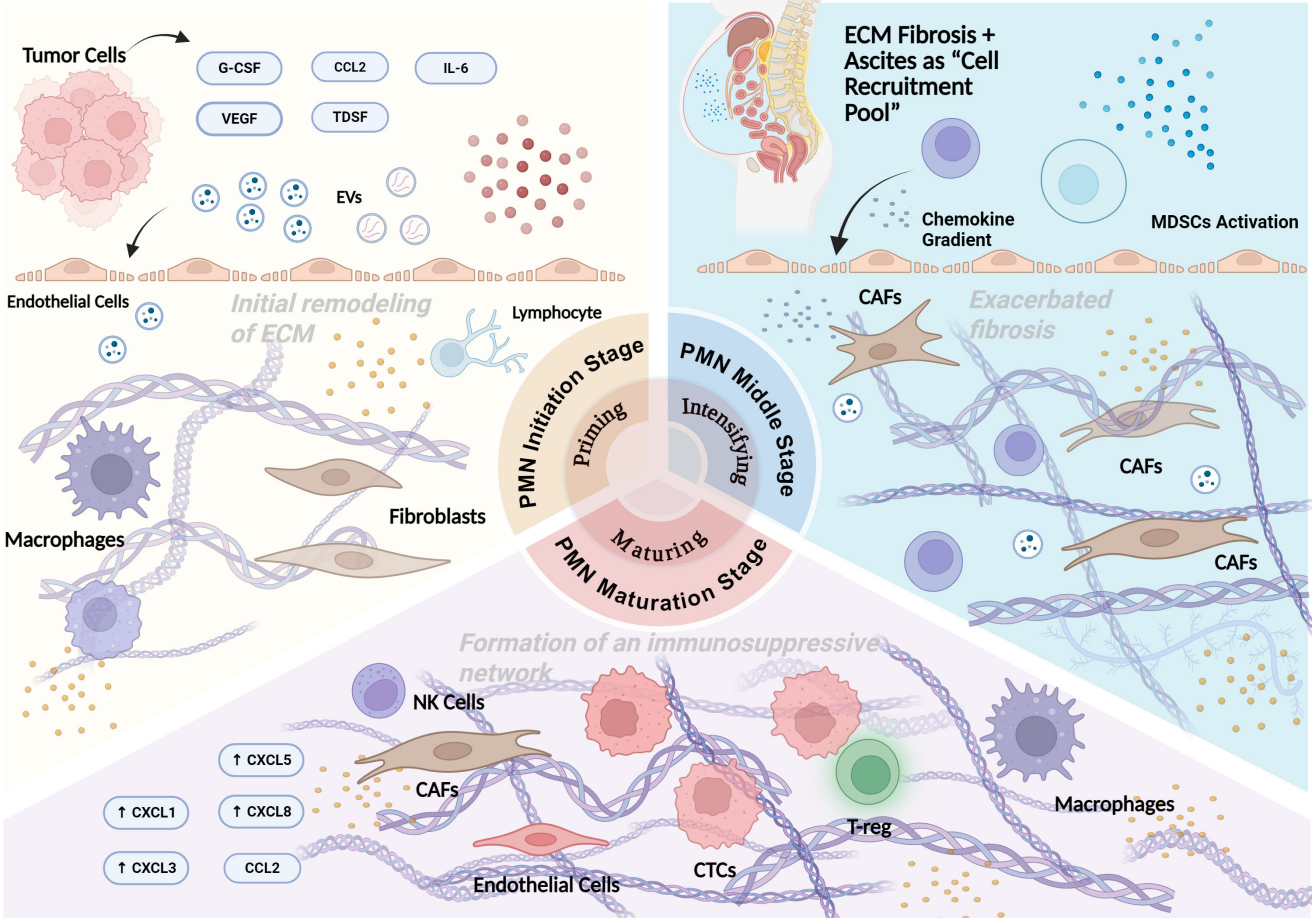
Spatiotemporal evolution of the PMN during gastric cancer metastasis. This figure delineates the sequential stages of PMN development—initiation, intensifying (middle stage), and maturation—showing how tumor-derived factors coordinate extracellular matrix (ECM) remodeling, recruitment and crosstalk of immune cells (e.g., macrophages, myeloid-derived suppressor cells (MDSCs), regulatory T cells (Tregs), natural killer (NK) cells) and stromal cells (e.g., cancer-associated fibroblasts (CAFs), endothelial cells). These integrated processes shape a favorable microenvironment that enables adhesion, extravasation, and colonization of circulating tumor cells (CTCs) at metastatic sites.

After tumor cells penetrate the serosa into the peritoneal cavity, ascites not only contains cytokines involved in disrupting mesothelial barriers but also enriches a large number of tumor-derived exosomes (65). These ascitic exosomes “reprogram” macrophages via carried miRNAs, polarizing them into a cancer-promoting phenotype that continuously secretes cytokines, forming an “exosome-immune cell-tumor cell” positive feedback loop to accelerate tumor cell invasion and colonization (66). During metastatic lesion development, as focusing factors coordinate stromal distribution, exosomes in ascites and body fluids continuously transmit related signals to precisely regulate extracellular matrix degradation and neovascularization timing (67). They coordinate stromal-tumor cell spatiotemporal distribution, optimize angiogenic factor secretion for metastatic blood supply and structural stability, ultimately completing the “primary detachment-peritoneal colonization-metastasis formation” dissemination chain (68). (**Figure 2**)

## 4 Application of organoid models in research on peritoneal metastasis of gastric cancer

### 4.1 Limitations of traditional two-dimensional culture models in gastric cancer research

Two-dimensional (2D) gastric cancer cell cultures serve for studying cell biology and preliminary drug screening, with advantages: easy operation, low cost, large-scale expansion. But their biological limitations have become a bottleneck, restricting gastric cancer research in precision and clinical translation (69). Firstly, 2D systems cannot recapitulate *in vivo* 3D gastric cancer structure and cell polarity. Gastric cancer cells grow as adherent monolayers on plastic dishes, losing key gastric gland characteristics such as glandular lumens, intercellular junctions (e.g., tight junctions), and polarized distribution (e.g., apical E-cadherin). This disrupts the cytoskeleton, activates signaling pathways (e.g., Hippo-YAP), and ultimately induces substantial differences between cultured cells and *in vivo* tumor cells (70).

Secondly, 2D models lack key gastric cancer microenvironment components and can’t simulate tumor-stromal crosstalk. Gastric cancer progression depends on microenvironments including fibroblasts, immune cells, vascular cells, and ECM (e.g., collagen) (71). However, 2D cultures have only single gastric cancer cells, lacking ECM’s mechanical/biochemical support and stromal cell cytokines, which stops accurate analysis of cancer cell-microenvironment crosstalk. E.g., they can’t simulate fibroblasts recruiting cancer cells via CXCL12 to promote invasion, nor reproduce ECM stiffness-induced EMT in gastric cancer (58, 72).

Furthermore, 2D cultures tend to cause the depletion of gastric cancer cell heterogeneity and distortion of functional phenotypes. Gastric cancer is a typical heterogeneous tumor, containing multiple subtypes such as cancer stem cells and differentiated tumor cells. However, the selection pressure in 2D cultures preferentially retains adherent cells with strong proliferative capacity. It gradually eliminates rare subtypes like cancer stem cells, leading to model homogenization that fails to reflect the heterogeneous characteristics of *in vivo* tumors (73, 74).

These limitations collectively indicate that 2D culture models can no longer meet the needs of current research on gastric cancer pathological mechanisms and precision therapy exploration. Thus, there is an urgent need for more physiologically relevant model systems to achieve breakthroughs. (**Table 1**)

**Table 1:** Comparison of the experimental models in solid organ cancer research.

Dimension	Cell Culture (2D)	Organoid Culture (3D)	Animal Models	PDX Models	References
**Core Materials & Setup**	Cancer cell lines grown in monolayer on culture plates.	Primary tumor cells/stem cells + Matrigel, forming organoids via 3D suspension culture.	Immunodeficient mice (e.g., NSG mice) + tumor cell/tissue implantation.	Patient-derived tumor tissue implanted into immunodeficient mice (e.g., NSG mice), preserving tumor heterogeneity.	(75, 76)
***In Vivo* Tumor Mimicry**	Low: Lack of ECM and 3D structure; poor recapitulation of tumor microenvironment.	High: Retains tumor architecture, cellular heterogeneity, and partial microenvironment (e.g., angiogenesis).	Moderate: Simulates tumor growth, metastasis, and immune responses, but species differences may affect results.	High: Preserves genetic features, drug response, and microenvironment of patient tumors.	(77, 78)
**Duration**	Short	Moderate	Long	Long	(79)
**Operational Complexity**	Low: Standardized protocols, minimal equipment required.	Moderate: Requires optimization of culture media and 3D conditions.	High: Involves animal husbandry, surgical implantation, and ethical approvals.	High: Requires fresh tumor tissue, surgical expertise, and long-term monitoring.	(80)
**Throughput & Cost**	High-throughput, low cost: Handles hundreds of samples simultaneously.	Moderate-throughput, moderate cost: Requires Matrigel and specialized equipment.	Low-throughput, high cost: Expensive animal maintenance and experimental consumables.	Low-throughput, extremely high cost: Dependent on patient samples and long-term upkeep.	(81, 82)
**Applications**	Basic mechanism studies (e.g., signaling pathways, drug toxicity screening).	Drug sensitivity testing, tumor heterogeneity research, and personalized medicine.	*In vivo* efficacy evaluation, metastasis studies, and immunotherapy validation.	Preclinical drug screening, biomarker discovery, and patient-specific therapy.	(83)
**Major Advantages**	Rapid, economical, and easy to scale; ideal for large-scale screening.	Accurate recapitulation of tumor microenvironment; long-term culture with retained heterogeneity.	Enables study of tumor-host interactions and dynamic processes *in vivo*.	Closest genetic mimicry of patient tumors; high predictive accuracy for clinical response.	(84, 85)
**Major** **Limitations**	Poor correlation with clinical outcomes due to lack of microenvironmental context.	Lack of intact immune system; potential loss of tumor features during long-term culture.	Species-specific drug response variations; ethical concerns.	Low engraftment rates (<20% for some tumor types), long timelines, and high costs.	(78, 82, 85, 86)

### 4.2 Background of organoid research and construction of gastric cancer organoids

The concept of organoids can be traced back to the early twentieth century. In 1907, Henry Van Peters Wilson first showed that dissociated sponge cells, when cultured *in vitro*, could spontaneously re-aggregate and self-organize to regenerate a complete, functional organism (87). This finding laid the foundation for researching the core self-organization property of organoids. Later, scientists explored cell separation and reaggregation related to organ repair using models like amphibian pronephros and chicken embryos (88, 89).

**Figure 3:**
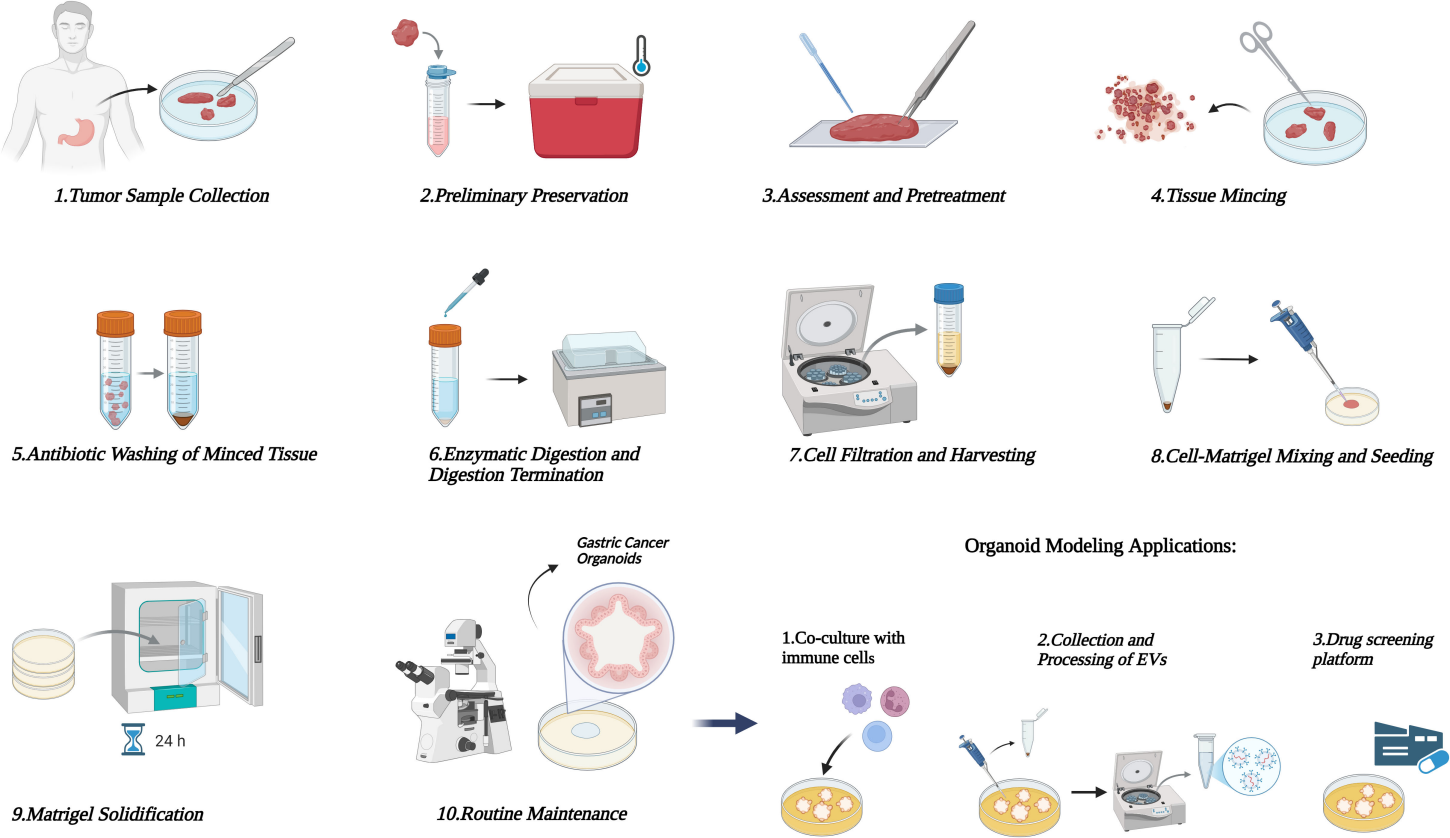
Standardized protocol for gastric cancer organoids culture. This figure illustrates 10 key steps of the standardized protocol for gastric cancer organoids culture, including tumor sample collection, preliminary preservation, assessment and pretreatment, tissue mincing, antibiotic washing of minced tissue, enzymatic digestion and digestion termination, cell filtration and harvesting, cell-Matrigel mixing and seeding, Matrigel solidification, and routine maintenance, providing a technical procedure reference for their *in vitro* construction and research.

The construction of gastric cancer organoids starts with the acquisition of patient tumor samples, typically collected from surgical resection specimens or endoscopic biopsy tissues. These samples are stored in ice-cold PBS containing antibiotics and transported at low temperature to preserve cell viability (71, 90). During laboratory processing, adipose and necrotic tissues are first removed, and the tumor tissue is cut into 1–2 mm pieces. After washing with antibiotic-containing buffer, the tissue pieces are enzymatically digested at 37 °C using a solution with collagenase and hyaluronidase. Under microscopic monitoring, digestion is stopped once cell clusters are released; the digested product is then filtered through a cell sieve, and cell pellets are collected by centrifugation (91, 92). Subsequently, the cells are gently mixed with basement membrane matrix (e.g., Matrigel) at an appropriate ratio on ice to avoid air bubbles. The mixture is inoculated as droplets into pre-warmed culture plates and incubated upside down to solidify the matrix (93). Finally, a specialized organoid medium containing cytokines such as EGF, Noggin, and R-Spondin 1 is added slowly to fully cover the matrix. With medium replacement, subculture is performed according to growth status, allowing long-term culture while maintaining tumor characteristics (94, 95). (**Figure 3**)

**Figure 4:**
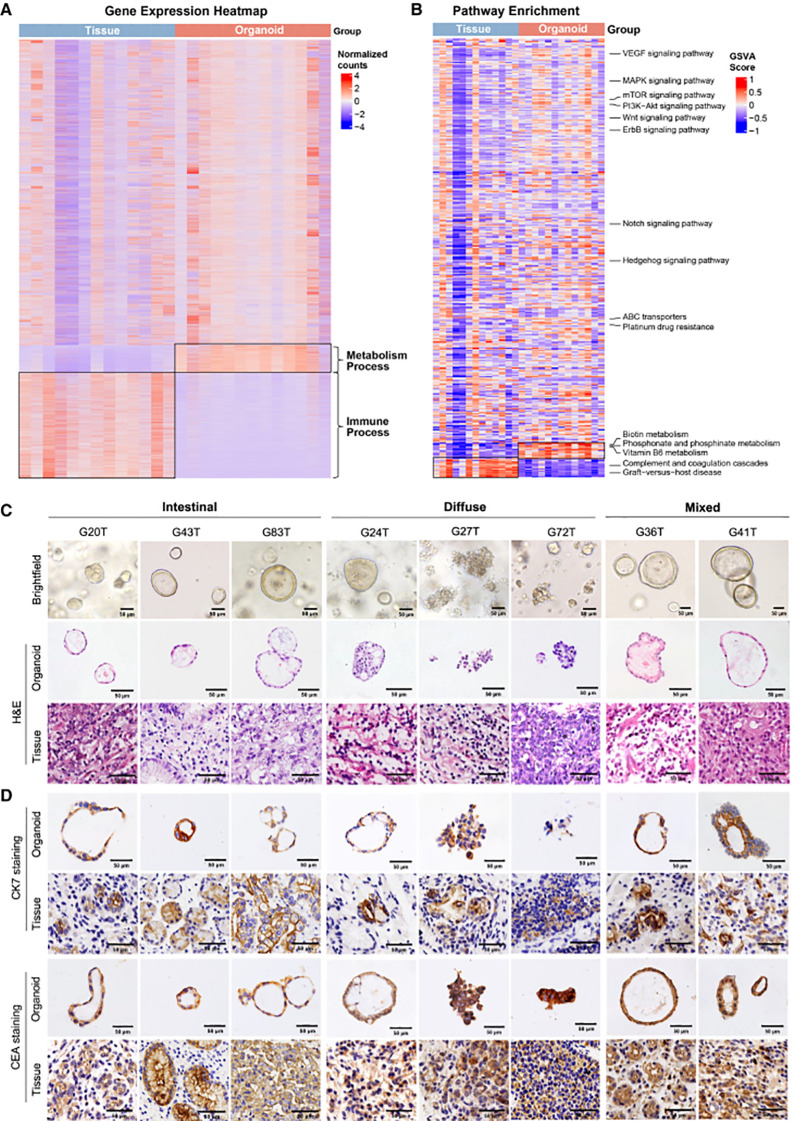
**(A–D)** Transcriptome and histopathology of GC organoids vs. primary GCs. This figure presents the heatmap and pathway analysis of differentially expressed genes, bright-field and H&E staining images, as well as immunohistochemical results of GC markers CK7 and CEA between organoids and their corresponding tumor tissues, with the scale bar indicated (86). Copyright 2024 Elsevier.

For example, a multi-dimensional analyze further compared the RNA sequencing results of organoids with their corresponding primary tumors, and found that the two exhibit a high degree of similarity in their gene expression patterns. Bright-field microscopy and H&E staining visually demonstrated the structural morphology of organoids, which is highly consistent with the histologic phenotypes of primary lesions. Immunohistochemical detection of protein markers (e.g., CK7, CEA) further confirmed that organoids retain the protein expression patterns of primary tumors. Collectively, these pieces of evidence confirm the successful establishment of the gastric cancer organoid model, laying a reliable foundation for simulating tumor biological behaviors (86) (**Figure 4**).

The development of patient-derived organoids (PDOs) has reshaped the field of personalized medicine, with a notable focus on advancing cancer research. Derived from human tissues, PDOs exist as three-dimensional (3D) cultures that preserve the genetic, phenotypic and architectural features of the original tumor. This trait makes them a more robust model for investigating tumor biology than conventional two-dimensional (2D) cultures. Recent progress in organoid technology has focused on refining protocols for PDO creation, as well as improving their growth rate, survival capacity and functional reliability. (**Table 2**)

**Table 2:** Organoid culture technologies comparison.

Technology Type	Principle	Major Applications	Main Advantages	Current Limitations	References
Static Culture	Cells or organoids grow embedded in ECM or culture medium under stationary conditions	Basic organoid cultivation and model establishment	Simple, easy operation; suitable for preliminary expansion and screening	Lack of vascularization and physiological microenvironment	(100, 101)
Hanging Drop Method	Cells suspended in small droplets self-aggregate into spheroids	Forming uniform spheroids simulating cell-cell interactions	Simple and efficient; good spheroid uniformity	Limited scalability and cell number	(102–105)
Dynamic Suspension Culture	Use of rotators or stirrers to keep cells/organoids suspended, improving nutrient and oxygen flow	Large-scale organoid expansion and culture	Improved oxygenation and nutrient supply; enhanced viability	Shear stress may harm cells; higher equipment costs	(106–109)
Magnetic Levitation	Cells tagged with magnetic nanoparticles are levitated by magnetic fields to assemble tissues	Rapid 3D cell assembly and organoid modeling	Provide a complex culture environment	Potential nanoparticle cytotoxicity; technology is relatively new and lacks standardization	(110–113)
Porous Microsphere Scaffolds	Cells seeded on porous microspheres providing 3D microenvironment support	Supporting cell adhesion, proliferation, and differentiation	Biomimetic 3D scaffold promotes cell growth and tissue assembly	High complexity and cost in preparation; Heterogeneous structure of microsphere scaffolds	(114–116)
Microfluidic Chip Method	Cells cultured in microfluidic devices under dynamic flow to mimic *in vivo* microenvironment	Provide a 3D growth environment; Precisely control the microenvironment of cells	High spatiotemporal control	Complex devices; high cost; technically demanding; limited scalability	(117–119)
Air-Liquid Interface (ALI) Culture	Cells cultured at the interface between air and liquid to promote polarization and differentiation	Disease modeling; Infection and Immunity	High physiological relevance	Size and lack of vascularization; Microenvironment simplification	(120, 121)
Bioprinting	Layer-by-layer 3D printing of cells and biomaterials to build complex organoid structures	High precision construction of complex 3D organoids	Highly controllable spatial architecture; precise multi-cell type placement	Technically complex; slow printing speed; maintaining cell viability during printing challenging	(122–126)
Hydrogel Embedding	Cells encapsulated in natural or synthetic hydrogels forming a biomimetic 3D extracellular matrix	Large-scale cultivation; Drug screening	Provide bioactive signals to support the directional differentiation of cells; Ease of operation	Standardization deficiency; Insufficient long-term stability	(107, 127, 128)

Techniques like microfluidic droplet encapsulation are used to direct the growth and organization of tumor cells, which leads to higher purity and success rates in organoid formation compared to traditional methods (96).

Additionally, using tailored culture media that mimic the *in vivo* microenvironment significantly improves the growth and maintenance of PDOs, helping them better reflect the tumor’s response to therapies (97). Furthermore, integrating advanced bioprinting technologies enables the precise spatial arrangement of cells within organoid cultures, which can enhance the physiological relevance of these models (98). Being able to generate organoids from various tissue types, including those from rare cancers, expands the potential applications of PDOs in drug screening and disease modeling, thereby paving the way for more effective and individualized treatment strategies (99). As these technologies continue to evolve, they offer promise for closing the gap between laboratory research and clinical applications, ultimately improving patient outcomes in oncology. (**Table 2**)

### 4.3 Advantages and limitations of organoid models in PMN research

Organoids in peritoneal metastasis research have distinct advantages, especially simulating dynamic tumor-peritoneal microenvironment interactions with high spatiotemporal resolution. As 3D structures from patient tissues, they closely mimic real tumor architecture and function, providing a more realistic platform for tumor-mesothelial interaction studies. For, example, to clarify the oncogenic effect of PTBP3, a research team took PDO as the core experimental model and conducted *in vitro* studies combined with gastric cancer cell lines. By virtue of their characteristics closely mimicking clinical tumor heterogeneity, PDO directly confirmed the enhancing effect of PTBP3 on tumor cell invasion and proliferation, providing highly credible clinically relevant evidence for the oncogenic function of this molecule. More importantly, PDO were used to construct xenograft models, serving as the core carrier for evaluating novel combined therapies (129).

Moreover, organoid models facilitate the dynamic monitoring of molecular events associated with PMN formation. Unlike conventional cell culture systems, organoids enable real-time observation of cellular responses to various stimuli, including pharmacological agents, and can also be used to assess how different microenvironmental conditions affect tumor behavior. In gastric cancer, malignant ascites-derived organoids (MADOs) have demonstrated that the liquid tumor microenvironment significantly enhances their colony formation capacity, indicating that crosstalk between exfoliated cancer cells (ECCs) and their surrounding milieu is critical for PMN development (130).

Interestingly, another study employed time-lapse photography to clearly capture the dynamic changes of individual malignant ascites-derived organoids (MADOs). Through this observation method, it was found that malignant ascites (MA) supernatant could significantly enhance the formation efficiency and size of MADOs by promoting cell division. The application of MADOs in this study provides important support for cancer research in terms of spatiotemporal resolution. Besides, in EVs research, the functional verification value of MADOs is particularly prominent. Initially, it was hypothesized that exosomes in MA might mediate tumor-promoting effects, but MADOs clearly ruled out this assumption through standardized control experiments. MADOs were co-cultured with exosomes, MA supernatant, and the supernatant fraction without exosomes, respectively. The results showed that the exosome group had no significant effect on organoid growth, while both the MA supernatant group and the exosome-depleted fraction group exhibited a strong growth-promoting effect (131). Therefore, the core value of organoid research lies not only in real-time capturing spatiotemporal dynamic changes of individual organoids, but also in accurately determining whether specific factors function in cancer development and their specific modes of action. Furthermore, it identifies the core regulatory role of key factors, providing direct experimental support for analyzing the mechanism of mutual collaboration between tumor cells and components of the liquid microenvironment.

Correspondingly, although organoids can mimic source tissues, they have major limitations. To begin with, they have cell self-organization problems—unable to accurately replicate the original organ’s dimensions (size, shape), cellular composition, phenotypic or molecular traits. More critically, current tech can’t make organoids with complex vascular networks supported by VSMCs, limiting their use in inflammation research. Because tissue inflammation needs vascularization to recruit *in situ* immune cells, even spinning bioreactors such as co-culturing MSCs with endothelial cells that form vascular-like networks haven’t fully solved this. Also, organoids have limited well-differentiated ECM components. While organoids rely on ECM-guided stable growth to recapitulate the characteristics of their source organ, insufficient ECM complexity results in weak immune responses. This, in turn, limits investigations into tumor infiltration and drug penetration—two key processes in peritoneal metastasis research (132).

Promisingly, relevant studies have offered insights into addressing these issues. First, the IOI-Chip microfluidic chip uses its baffle-well structure to trap small quantities of PDOs and autologous immune cells, enabling long-term co-culture. It also quantifies key PMN-associated cytokines *in situ* via microbead-based immunofluorescence (133). Second, the cancer organoid-on-a-chip can co-culture mesenchymal stromal cells, cancer-associated fibroblasts, PDOs, and peripheral blood mononuclear cells (PBMCs). MSCs improve PDO culture success rates and induce monocyte differentiation into tumor-associated macrophages, while the multi-layer chip further mimics the dynamic PMN environment and enables high-throughput drug screening. These incorporated technologies effectively tackle the drawbacks of organoids, delivering a more precise *in vitro* model for PMN study (134).

In summary, organoid models represent a powerful tool in PMN research, providing high-resolution insights into tumor-stroma interactions and enabling the dynamic monitoring of molecular events that drive peritoneal metastasis. It also facilitate real-time observations positions organoids as a critical asset in the quest to unravel the intricacies of cancer metastasis and to identify novel therapeutic interventions.

## 5 The role of extracellular vesicles in PMNs

### 5.1 Biological characteristics and functions of EVs

Extracellular vesicles (EVs) are heterogeneous membranous structures released by diverse cell types including tumor cells, and comprise subsets such as exosomes (30–150 nm), microvesicles (100–1,000 nm), apoptotic bodies (>500 nm) and other less characterized classes. In particular, exosomes derive from the endosomal compartment and form through the inward budding of the membrane. This process results in the formation of multivesicular bodies that then fuse with the plasma membrane to release exosomes into the extracellular space (135). The cargo of EVs contains a variety of biomolecules, including proteins, lipids, mRNAs, and non-coding RNAs, these molecules reflect the physiological state of their parent cells and can influence recipient cells once taken up (136, 137). EVs’ specific molecular composition varies greatly based on their cellular origin and the pathological context, offering insights into their functional roles in intercellular communication and disease progression.

Within the framework of cancer, EVs function as important mediators of cell-cell signaling, facilitating the conveyance of bioactive molecules capable of regulating the tumor microenvironment. The ability of EVs to encapsulate and transport these diverse molecular cargos makes them potent tools for both therapeutic applications and as biomarkers for cancer diagnosis and prognosis. Their unique properties, including biocompatibility and low immunogenicity, position EVs as promising candidates for drug delivery systems, particularly in targeting tumor cells while sparing healthy tissues (138, 139).

Overall, the study of EVs is rapidly evolving, with ongoing research aimed at elucidating their complex roles in health and disease, particularly in the context of cancer. The exploration of EVs not only advances our understanding of tumor biology but also opens new avenues for therapeutic interventions. These interventions leverage the natural properties of EVs to enable targeted delivery of drugs and genetic materials (136). As research progresses, it is anticipated that the clinical utility of EVs will expand, leading to innovative strategies for cancer treatment and improved patient outcomes.

### 5.2 EVs-mediated immune suppression

EVs serve as key mediators of immune suppression in the tumor microenvironment, especially in cancers like gastric cancer and glioblastoma. A major mechanism for their immunosuppressive effects lies in delivering immune inhibitory molecules including programmed cell death ligand 1 (PD-L1), transforming growth TGF-β, and microRNAs that regulate the expression of various immune-related genes in recipient cells. PD-L1, a well-recognized immune checkpoint molecule, is expressed not only on tumor cells but also on EVs derived from these cells. The PD-L1 carried by EVs can bind to PD-1 receptors on T cells, which inhibits T cell activation and proliferation and thereby facilitates tumor immune evasion (140, 141). For example, exosomal PD-L1 which derived from gastric cancer cells binds to PD-1 receptors on T cells. Although exosomal PD-L1 is not a soluble free form, its membrane-bound property still mediates direct inhibition of T cell activation via this canonical immune checkpoint interaction, impairing T cells’ anti-tumor efficacy. More crucially, exosomal PD-L1 can also drive the activation and expansion of myeloid-derived suppressor cells by activating the IL-6/STAT3 signaling pathway (142). Additionally, TGF-β, another critical immunosuppressive factor, is often found in EVs and can induce the differentiation of Tregs while inhibiting effector T cell functions. This dual action results in a more pronounced immunosuppressive environment that allows tumors to thrive despite the presence of an active immune system (143, 144). Interestingly, studies have demonstrated that Epstein-Barr virus (EBV)-infected gastric cancer cells secrete increased amounts of exosomes. These exosomes markedly suppress the maturation of monocyte-derived dendritic cells (moDCs)—a phenomenon characterized by a significant reduction in the proportion of dendritic cells positive for the maturation marker CD86.The key mechanism underlying this suppressive effect may be associated with the microRNAs carried by exosomes (145).

**Figure 5:**
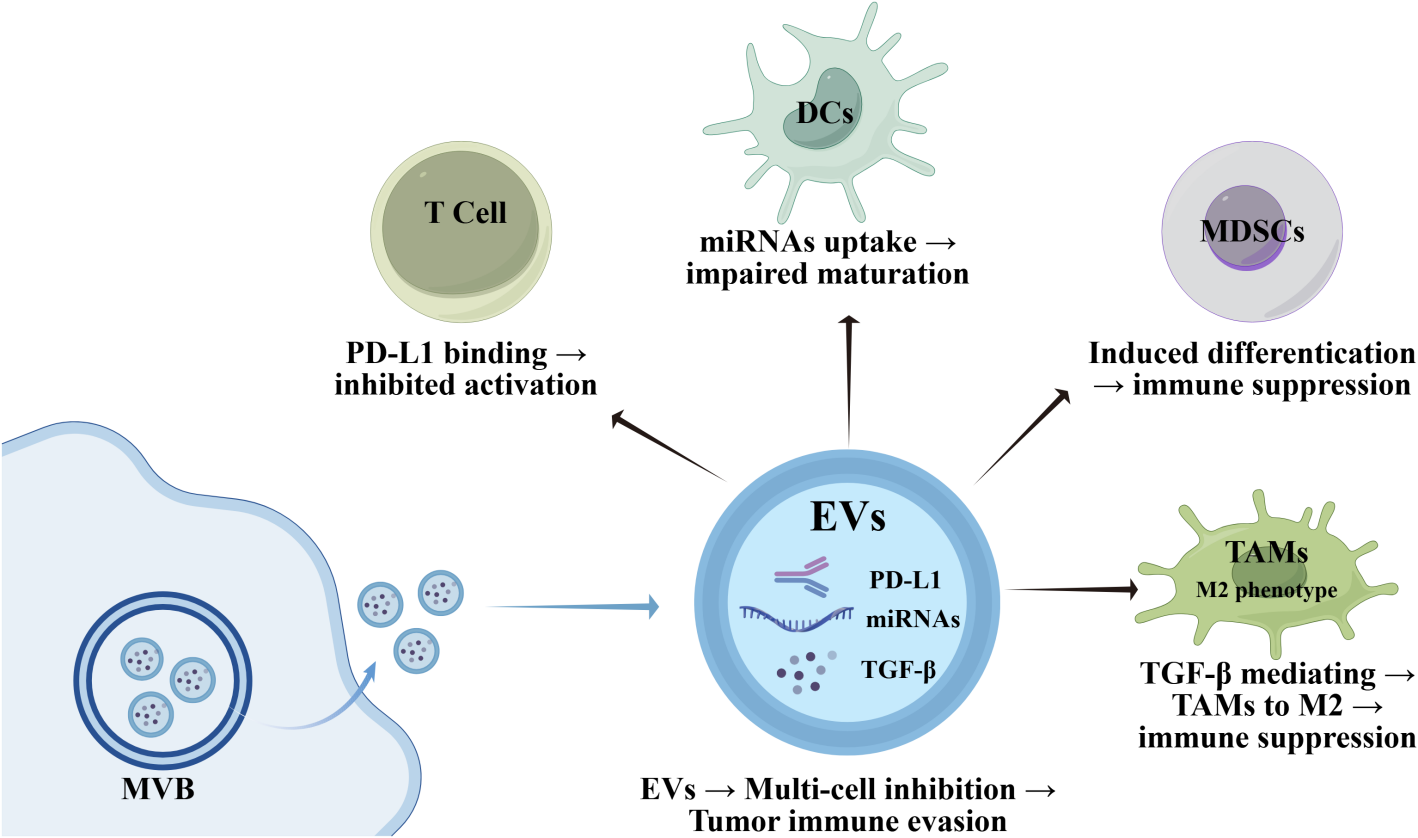
Mechanism of immune cells in tumor immune evasion mediated by extracellular vesicles. EVs derived from multivesicular bodies (MVB) carry molecules such as PD - L1, microRNAs (miRNAs), and transforming growth factor - β (TGF - β), and promote tumor immune evasion by inhibiting immune responses in multiple dimensions: binding to molecules on the surface of T cells to inhibit their activation; impairing the maturation of dendritic cells (DCs) after being taken up by them; inducing the differentiation of myeloid - derived suppressor cells (MDSCs) to exert immunosuppressive effects; mediating the polarization of tumor - associated macrophages (TAMs) toward the M2 phenotype via TGF - β, thereby generating immunosuppressive effects.

Moreover, tumor-derived EVs have been shown to promote the differentiation and activation of MDSCs, which are known to inhibit T cell responses and promote tumor growth (146, 147). Similarly, TAMs, which can adopt an immunosuppressive M2 phenotype in response to signals from tumor cells, are influenced by the cargo delivered via EVs. This interaction not only enhances their immunosuppressive capabilities but also aids in the maintenance of a pro-tumor environment (148) (**Figure 5**).

### 5.3 Gastric cancer-derived EVs orchestrate organ-specific metastasis through integrin-mediated targeting

Gastric cancer (GC)-derived EVs mediate organ-specific metastasis primarily through integrin-dependent targeting mechanisms, where surface integrins αvβ3 and α6/αx dictate distinct organotropism. Integrin αvβ3 on GC-derived EVs facilitates peritoneal colonization by recognizing and binding to the RGD motif of periostin (POSTN) in the peritoneal extracellular matrix. This interaction activates downstream FAK/Pyk2 signaling, which in turn enhances cancer cell adhesion and migration (36, 149) (36, 149). This interaction not only provides physical anchorage but also recruits macrophages via POSTN-integrin αvβ3 engagement, initiating the formation of an immunosuppressive pre-metastatic niche (52).

In contrast, integrins α6 and αx confer gastric-specific uptake of exosomes by recipient cells. EVs derived from gastric epithelial cells and GC cells are preferentially internalized by gastric epithelial cells and macrophages through clathrin-mediated endocytosis, a process strictly dependent on α6/αx expression (150, 151). This selective uptake ensures targeted delivery of exosomal cargoes to gastric tissues, as validated by reduced efficiency in α6/αx-silenced models (152, 153).Other integrins also contribute to organotropic metastasis: exosomal α6β4 and α6β1 are associated with lung metastasis, while αvβ5 promotes liver colonization (36).

## 6 The promotion of spatiotemporal resolution technology in PMN research

In gastric cancer research, spatiotemporal resolution technologies complement prior organoid modeling and EVs studies, jointly deepening our understanding of the PMN. Prior organoid modeling simulates 3D structure and cell interactions *in vitro*, while EVs research reveals their role in PMN regulation and metastasis, yet both have limitations. Organoids fail to capture *in vivo* PMN’s spatiotemporal dynamics, and EVs studies can’t trace vesicle origins or real-time regulation at single-cell level. Spatiotemporal technologies address these research gaps, among which single-cell sequencing, hailed as a pivotal tool, has revolutionized our understanding of the cellular heterogeneity during the metastatic process. Specifically, single-cell sequencing profiles individual cells to characterize the diverse cellular composition and functional states of the PMN. These findings refine organoid models using single-cell data and complement EVs research by identifying functionally active EV-secreting cells and tracking their dynamic crosstalk with PMN-resident target cells.

### 6.1 Single-cell sequencing in the analysis of PMN

The application of single-cell sequencing technologies has transformed our comprehension of cellular heterogeneity in the PMN, especially in the setting of cancer metastasis. Single-cell RNA sequencing enables detailed profiling of individual cells, uncovering the varied cellular compositions and functional states that underpin the PMN microenvironment. For instance, a single-cell transcriptomic analysis of 191,987 cancer and immune cells from ascites of 35 patients demonstrated that pro-angiogenic monocyte-like dendritic cells accumulate during the progression of gastric cancer peritoneal metastasis. This study also characterized the therapy-induced evolution of monocyte-like DCs, regulatory T cells, and proliferative T cells, and identified high-plasticity gastric cancer (GC) cell clusters that transition via autophagy-dependent paligenosis (154). Furthermore, cancer-associated fibroblast subpopulations characterized by high INHBA and FAP coexpression accumulated in a stage-wise manner. Single-cell comparisons of PDOs with primary tumors revealed both inter- and intralineage similarities and distinctions. Spatial transcriptomics, orthogonal validation across independent bulk RNA-sequencing cohorts, and functional demonstration were employed to supplement these findings (155).To investigate early gastric cancer (EGC) and its tumor microenvironment, researchers employed single-cell RNA sequencing to build an atlas of 184,426 high-quality cells from multi-stage clinical samples. Through this approach, they recognized 8 cell lineage states. As the disease progressed, epithelial meta-clusters declined whereas T&NK cells, B cells, plasma cells, fibroblasts, myeloid cells, and endothelial cells increased. Specific subclusters—including epithelial subclusters (MSCs, PMC-like cells, proliferating cells), T-cell subclusters (Treg, CCR7+ naive cells, etc.), and endothelial subclusters (IL-33+ Venous-1)—were elevated in EGC (156).

### 6.2 Spatial transcriptomics in the analysis of PMN

In addition to characterizing cellular heterogeneity, spatial transcriptomics has emerged as a complementary technique for enhancing our understanding of the pre-metastatic niche (PMN). Specifically, it provides spatial context for the cellular interactions identified via single-cell sequencing. By integrating spatial transcriptomics with scRNA-seq, researchers can pinpoint the localization of specific cell types and their associated signaling pathways within the PMN (157). For example, diffuse-type gastric cancer (DGC) originates from intramucosal lesions containing differentiated, non-proliferative signet ring cells (SRCs). It progresses to aggressive tumors by suppressing the differentiation of DGC cells. To investigate the driving molecular alterations, the researchers performed spatial transcriptomic analysis on tumors from patients with hereditary DGC at different stages and conducted functional assays in a CDH1-knockout (KO) human gastric organoid model (158). In studies examining the association between incomplete gastric intestinal metaplasia (Inc IM) and gastric cancer (GC), the joint application of spatial transcriptomics, subtype-specific organoids, and single-cell RNA sequencing played a crucial role in confirming Inc IM’s preneoplastic function. Spatial transcriptomics localized Inc IM to deep antral gland cells within GIM/GC tissues; it also identified Inc IM’s hybrid gastric-small-large intestinal transcriptional signature across differentiated cells and stem/progenitor cells, while initially establishing its molecular identity. Subtype-specific GIM organoids replicated Inc IM’s phenotype and lineage plasticity; analyses of DNA methylation and chromatin accessibility revealed key intergenic hypermethylation in Inc IM and distinguished it from complete GIM. Single-cell RNA sequencing supplemented data related to Inc IM’s differentiation (159). Interestingly, a research team established seven robust gastric cancer-specific meta-programs (MP1–MP7) using single-cell RNA sequencing and spatial transcriptomics techniques. Among these, MP7 exhibits the highest malignancy; it is surrounded by an immune-locked microenvironment and spatially associated with myofibroblasts (myCAFs). MP7 drives the conversion of fibroblasts into myofibroblasts (myCAFs) via the GDF15/TGFBR2 signaling pathway and inhibits the infiltration of CD8+ T cells. This creates an immune-deprived microenvironment surrounding MP7, which in turn results in poor efficacy of immunotherapy. In contrast, RSPO3 derived from myCAFs can up-regulate EGR1 expression in tumor cells, promoting the conversion of gastric cancer cells into the MP7 state and thereby enhancing the tumor’s invasiveness and drug resistance (160).

In conclusion, the application of single-cell sequencing technologies, coupled with spatial transcriptomics, has provided unprecedented insights into the cellular heterogeneity and functional dynamics of PMNs within the premetastatic niche.

### 6.3 *In vivo* imaging techniques for dynamic monitoring of PMN formation

The utilization of advanced *in vivo* imaging techniques, particularly multiphoton microscopy, has revolutionized our understanding of the dynamic processes involved in the formation of the PMN in gastric cancer. Multiphoton microscopy allows for the real-time tracking of EVs within living organisms, providing insights into their distribution and interaction with immune cells in the TME (52, 161).

For instance, in studies focused on the dynamic tracking of mesenchymal stem cell-derived extracellular vesicles (MSC-EVs), two-photon intravital microscopy (TP-IVM) serves as a pivotal technological pillar. By leveraging a custom-built abdominal imaging window in living animals and integrating it with lineage tracing approaches, this technique enables high-resolution *in vivo* imaging with distinct advantages. First, it allows clear visualization and tracking of the *in vivo* migration trajectory of EVs, thereby confirming whether EVs successfully reach their target sites of action. Second, it facilitates real-time dynamic observation of the activation and proliferation of specific cell populations after EV treatment, offering intuitive insights into the regulatory effects of EVs on target cells (162).Apart from that, at the *in vivo* research level, the research team directly visualized the dynamic processes of EV release from tumor cells and EV uptake by microenvironmental cells in the brain using two-photon intravital microscopy. Subsequent detection of the aforementioned target cells isolated from the brains of tumor-bearing animals revealed a significant increase in miR-21 levels and a notable decrease in c-Myc mRNA levels, further confirming the molecular regulatory role of EVs *in vivo (*162). As a newly developed technology, fluorescence nanoparticle tracking analysis (FL-NTA) enables accurate EV quantification by detecting EV-associated proteins. Combined with specific EV marker immunolabeling, it simultaneously acquires EV concentration, particle size distribution and surface phenotype in heterogeneous solutions, serving as an innovative tool for comprehensive EV characterization. For example, in non-small cell lung cancer (NSCLC) research, it has been used for systematic phenotyping of patient-derived BALF-EVs, showing advantages for pure EV isolates (163).

Although multiphoton microscopy serves as a core tool for *in vivo* imaging and enables high-resolution visualization of cellular/tissue structure, dynamics, and functions, it has a key limitation that compromises research reliability: the light illumination it depends on induces phototoxicity. Though the academic community has long known phototoxicity may distort experimental results, this issue has remained unresolved due to unclear global molecular consequences of intracellular damage. Yokoi et al. further confirmed this limitation using 3D organoids of small intestinal epithelial cells as a model combined with RNA sequencing: low-dose light irradiation, though not causing obvious morphological damage, already induced abnormal expression of genes related to reactive oxygen species response, metabolism, and immunity; high-dose light not only triggered cell apoptosis but also disrupted the structural formation ability of intestinal organoids and the granule secretion function of Paneth cells, with only drug efflux function unaffected. It is clearly revealed that the hidden phototoxicity risk of photon microscopy beneath its high-resolution advantages, providing an important basis for avoiding phototoxic artifacts and ensuring result accuracy in subsequent experiments (164).

In summary, these advances collectively lay the groundwork for advancing the trinity of future directions emphasized in our research: multi-model integration, multi-omics technologies, and clinical translation, which together form a cohesive research framework. Specifically, multi-model integration synthesizes *in vitro* organoids and *in vivo* imaging to better recapitulate PMN’s inherent complexity; multi-omics technologies focus on uncovering the molecular networks underpinning crosstalk between PMNs, EVs, and immune cells; and clinical translation seeks to translate these mechanistic findings into actionable targeted therapies. As we continue to unpack the complexities of PMN biology, this trinity framework will synergistically deepen our understanding of gastric cancer metastasis and refine therapeutic strategies, ultimately leading to improved outcomes for patients.

## 7 Potential therapeutic targets and future research directions

### 7.1 Therapeutic strategies of targeting PMN and reversing EVs-mediated immunosuppression

Therapeutic reprogramming of the PMN is crucial, as timely disruption of its soil-ready microenvironment can prevent metastatic seeding and transform incurable relapse into a preventable event.

EVs play a critical role in intercellular communication and can carry various molecular signals that influence the behavior of recipient cells and immune cells. In gastric cancer, EVs derived from tumor cells have been implicated in promoting MDSCs activation and skewing cytokine production towards an immunosuppressive profile, thereby facilitating tumor progression and metastasis (165). This suggests that targeting exosome-mediated pathways may represent a viable therapeutic approach to mitigate the immunosuppressive effects of in gastric cancer. One of the promising strategies involves the use of EVs inhibitors, such as GW4869, which have shown potential in modulating the immunosuppressive tumor microenvironment (166).Furthermore, small interfering RNAs (siRNAs) targeting RAB27A or nSMase2—key enzymes in extracellular vesicle (EV) biogenesis—further validate this strategy. Specifically, they reduce the levels of immunosuppressive cargo (TGF-β, PD-L1) in peritoneal fluid by 40–60% and reverse T cell anergy (167). More importantly, for EV-mediated niche remodeling, integrin αvβ3 antagonists such as Dis Ba-01 (500 nM) disrupt EV adhesion to fibronectin-rich peritoneal extracellular matrix by 70%, inhibiting subsequent uptake by mesothelial cells and macrophage polarization toward the M2 phenotype (168).

In addition to EVs inhibitors, the combination of immune checkpoint inhibitors, particularly anti-PD-1 monoclonal antibodies, has emerged as a critical strategy in the management of gastric cancer with peritoneal metastasis. In gastric cancer, the expression of PD-L1 has been associated with T-cell exhaustion and impaired anti-tumor immunity Preclinical studies have shown that targeting this axis with anti-PD-1 antibodies can reinvigorate T-cell responses, leading to enhanced tumor control. For example, in murine models of peritoneal metastasis, treatment with anti-PD-1 antibodies not only restored T-cell function but also significantly reduced tumor burden and improved overall survival (169). Encouragingly, another cancer study showed that advanced papillary and anaplastic thyroid cancers are resistant to standard therapies, and anti-PD-1 therapy is only effective in a small subset of patients. This study demonstrated that extracellular vesicles derived from M2 macrophages suppress METTL3 expression in cancer cells via miR-21-5p. Reduced METTL3 expression promotes the demethylation of CD70 mRNA, which inhibits CD70 mRNA degradation and subsequently increases CD70 protein levels. This, in turn, elevates the abundance of immunosuppressive regulatory T (Treg) cells and terminally exhausted T cells, ultimately inducing resistance to anti-PD-1 therapy. *In vivo* experiments demonstrated that combining the monoclonal antibody cusatuzumab to block CD70 could reverse this drug resistance (170).

In conclusion, the synergistic effects of combining EVs inhibitors with immune checkpoint blockade could potentially amplify therapeutic outcomes by disrupting the immunosuppressive microenvironment. Such combinatorial approaches are currently under investigation in clinical trials, aiming to establish a more effective treatment paradigm for patients and presenting a promising avenue for improving therapeutic efficacy in this challenging clinical scenario.

### 7.2 Next-generation EVs applications in gastric cancer: from liquid biopsy biomarker discovery to engineered drug delivery systems

EVs act as pivotal mediators of PMN formation and immunosuppression. Thus, advancing EVs detection technologies and developing strategies for early identification as diagnostic biomarkers is critical for improving GC patient survival (171). For example, liquid biopsy-based EVs analysis has emerged as a promising tool for early metastatic risk stratification. A meta-analyses of 12 cohort studies (n = 1,892 patients) demonstrate that circulating EVs-derived RNA panels (encompassing miRNAs and lncRNAs distinguish early-stage GC from healthy controls with a pooled AUC of 0.879, outperforming traditional serum markers (CA19-9, CEA) by 15–20% (172). Complementing RNA biomarkers, serum EV-associated proteins (GSN, HP, ORM1, APOA1, and TTR) form a 5-protein signature that achieves an AUC of 0.80 in independent validation cohorts, further enhancing diagnostic specificity for peritoneal metastasis (173). Moreover, In practical applications, to achieve early noninvasive diagnosis of colorectal cancer, a four-phase study was conducted. Through high-throughput transcriptome profiling of EVs and validation by RT-qPCR, the study identified 17 molecules significantly upregulated in CRC, 7 of which could effectively distinguish healthy controls from precancerous lesions. Two diagnostic panels developed based on these molecules exhibited high sensitivity and specificity in early CRC detection, with diagnostic performance outperforming the fecal occult blood test. It further confirmed that EVs serve as key reservoirs of CRC-associated molecules and a highly promising source of biomarkers (174). Technological advancements, such as aptamer-functionalized microfluidic chips, now enable sensitive and specific EV quantification (detection limit: 10² EVs/mL) with >90% capture efficiency—overcoming the limitations of conventional ultracentrifugation and facilitating the early detection of pre-metastatic niche initiation (175).

In addition, EVs have emerged as highly promising natural nanocarriers for cancer targeted therapy, attributed to their inherent superior biocompatibility and low immunogenicity, and these are unparalleled by synthetic carriers. The lipid bilayer structure of EVs can efficiently load various therapeutic cargos (e.g., chemotherapeutics, small interfering RNAs) while protecting them from enzymatic degradation (176). It was revealed that human umbilical cord mesenchymal stem cell-derived small extracellular vesicles (hucMSC-sEVs) inhibit gastric cancer progression by delivering anticancer microRNAs. Naturally enriched with anticancer miRNAs, hucMSC-sEVs contain the novel miR-13896 which suppresses proliferation, induces apoptosis, and blocks metastasis by targeting the ATG2A-mediated autophagy pathway. Engineered hucMSC-sEVs enriched with miR-13896 via electroporation were constructed, exhibiting efficient tumor targeting *in vitro* and *in vivo* and significantly inhibiting gastric cancer cell growth and migration (177). To address the key limitation of poor tissue retention of EVs, a research team developed an injectable EVs hydrogel-based cancer vaccine. Upon *in vivo* injection, the vaccine undergoes *in situ* gelation and remains stable for over 4 weeks. As a long-acting depot, it sustains the release of tumor antigens encapsulated in EVs, enabling prolonged modulation of dendritic cells and efficient activation of tumor-specific CD8+ T cells. Notably, it induces significantly stronger antibody responses compared to free EVs (178). Furthermore, Chimeric antigen receptor T (CAR-T) cell therapy, a cutting-edge cell-based immunotherapy for resistant cancer, has achieved commercial clinical application with proven efficacy. B, its clinical translation is severely hindered by the TME and adverse effects like cytokine release syndrome (CRS). As natural intercellular communication carriers, EVs possess the capacity to cross biological barriers and deliver functional molecules. Their unique immunomodulatory properties offer innovative solutions to overcome the limitations of CAR-T therapy (179).

Nevertheless, the clinical translation of EV-based therapy still faces formidable challenges: the recovery rate of EVs isolated by traditional ultracentrifugation is less than 50%, and they are prone to contamination by impurities such as lipoproteins. Even combined with polymer-based precipitation, it is difficult to fully meet the requirements of clinical-grade purity (180). There is no unified global standard for EV isolation and characterization, resulting in poor data reproducibility among different studies and seriously hindering technical promotion (181). But fortunately, with the continuous advancement of microfluidic separation technology, surface modification engineering, and automated production platforms, these limitations are being gradually addressed, laying the foundation for EVs to become core tools in precision oncology and realize the transformation from basic research to clinical application (182).

### 7.3 The potential of organoid models in personalized therapy

The emergence of patient-derived organoids (PDOs) has transformed the landscape of personalized medicine, especially in the field of oncology. One key advantage of organoid technology is its capacity to support drug sensitivity testing. This capability enables clinicians to tailor treatment regimens based on the unique response profiles of individual tumors. For instance, research has shown that PDOs can reliably forecast patient responses to various chemotherapeutic agents, thereby offering valuable guidance for clinical treatment decisions (183, 184). This predictive capacity is especially vital in gastric cancer, where conventional therapeutic approaches often fall short owing to the biological heterogeneity of tumors. By utilizing PDOs, clinicians can identify patients who are likely to derive benefit from specific drugs, thereby optimizing therapeutic outcomes and reducing unnecessary adverse effects.

In addition to drug screening, the advancement of organoid-immune co-culture systems has emerged as a significant breakthrough in personalized therapy. These systems facilitate interactions between organoids and immune cells, thereby mimicking the tumor microenvironment more precisely than traditional *in vitro* models. This co-culture system is vital for understanding the complex dynamics between tumors and the immune system, including how tumors escape immune surveillance and how immune responses can be leveraged for therapeutic gain (185, 186). The ability to model the immune landscape within organoids not only enhances our understanding of tumor biology but also paves the way for developing personalized immunotherapeutic strategies that are tailored to the specific immune profiles of patients.

Moreover, the potential for PDOs to serve as living biobanks is noteworthy. By establishing organoid libraries from diverse patient populations, researchers can perform high-throughput drug screenings and identify novel therapeutic targets across multiple cancer types. This biobanking approach not only accelerates drug discovery but also enables the investigation of rare cancers and cancer subtypes—entities that are often underrepresented in clinical trials (187, 188).

As organoid technology continues to evolve, it is expected that these models will play an increasingly pivotal role in the shift from traditional to personalized medicine, and serve as a solid platform for both basic research and clinical applications. The integration of organoid models into personalized therapy marks a major leap forward in cancer treatment. Their capacity to accurately mirror the biological features of tumors, predict drug responses, and support the study of tumor-immune interactions establishes them as key tools in the pursuit of tailored cancer therapies.

## 8 Conclusion

GCPM remains a core barrier to improving the prognosis of patients with advanced gastric cancer, and its lethality stems from insufficient understanding of the biological mechanisms underlying the PMN. As a dynamic microenvironment, the PMN is the key link in the “pre-activation” of the peritoneum to accommodate metastatic cell.

This study clarifies the spatiotemporal developmental framework of the PMN, defining it as a regulable system with stage-specific evolution. The PMN initiates with extracellular matrix remodeling and stromal cell activation, progresses through fibrotic transformation and recruitment of immunosuppressive cells, and ultimately matures into a stable microenvironment that supports the adhesion and proliferation of circulating tumor cells (CTCs). This description moves beyond previous isolated molecular observations, clarifies the dynamic nature of the PMN, and identifies key transition nodes such as myeloid cell infiltration and upregulation of matrix proteins, providing a theoretical basis for targeted intervention. Meanwhile, the research further establishes extracellular vesicles (EVs, especially tumor-derived exosomes) as core regulators of PMN formation, noting that EVs not only carry immunosuppressive substances to impair anti-tumor immunity (e.g., inhibiting T-cell function, inducing macrophage polarization toward a pro-tumor phenotype) but also achieve peritoneal tropism through surface integrins, establishing a bridge between primary tumors and distant PMNs. Additionally, it also emphasizes the transformative value of technological breakthroughs: patient-derived organoids (PDOs) can recapitulate the structural and cellular heterogeneity of primary gastric tumors, overcoming the limitation of 2D cultures that fail to simulate PMN dynamics; spatiotemporal resolution tools such as single-cell sequencing, spatial transcriptomics, and intravital microscopy can reveal the cellular heterogeneity of the PMN, resolve spatial signaling gradients, and track EV migration in real time, which successfully unlocking previously unobservable dynamic regulatory processes.

Key limitations in current research are also identified, which are consistent with the general challenges in organoid and cancer microenvironment research. On the one hand, although PDOs have physiological relevance, they lack functional vascular networks, which not only hinders the simulation of key PMN maturation processes (such as inflammatory cell recruitment and nutrient exchange) but also reduces the reliability of drug penetration studies; PDOs with *in vivo* physiology remains inadequate, as PDOs cannot replicate the complex mechanical signals of the peritoneal cavity or mesothelial cell plasticity. On the other hand, organoid models need standardization to accurately replicate the human peritoneal microenvironment, which is a critical prerequisite for ensuring their reliable clinical translation. The standardization of organoid models is crucial for accurately replicating the human peritoneal microenvironment and serves as a critical prerequisite for ensuring their reliable clinical translation. Organoid standardization mainly comprises two core dimensions: a standardized operational system and a standardized quality assessment system. The operational system covers tissue collection and preprocessing, organoid culture, passage, cryopreservation, and disposal of spent organoids. Notably, with the increase in organoid passage number, the genetic variation concordance between organoids and primary gastric cancer tissues gradually decreases. The quality assessment system includes morphological evaluation, biological function identification, and molecular biological identification. Morphological evaluation employs light microscopy observation, histopathological examination, and artificial intelligence-based analysis. Biological function identification focuses on key biological characteristics and substance metabolism, with indicators such as organoid ATP content detectable by chemiluminescence. Molecular biological identification involves lineage-specific biomarker detection and genetic stability verification. The combined application of advanced technologies such as single-cell RNA sequencing and spatial transcriptomics can more accurately confirm that organoids have high transcriptional homology with primary tissues.

Research on extracellular vesicles (EVs) faces technical bottlenecks in sensitive and high-throughput detection. Existing methods fail to capture the phenotypic heterogeneity of EVs, which limits the validation of EV-derived biomarkers for the early diagnosis of GCPM. Advancing the development of this field and the translation of medical applications primarily focuses on two key aspects. First, multi-model integration. Combining PDOs with microfluidic chips to recapitulate peritoneal fluid dynamics and vascular-like networks, or integrating PDOs with animal models to validate PMN mechanisms *in vivo*, bridging the gap between *in vitro* mechanistic studies and clinical practice; second, multi-omics analysis of EV-PMN-immune cell crosstalk. Using single-cell proteomics and spatial metabolomics to uncover new targets beyond existing immune checkpoints or EV biogenesis pathways.

In summary, this review points out that overcoming current limitations via model integration and a translation-oriented approach will not only deepen the understanding of GCPM pathogenesis but also bridge biological insights with clinical treatment. Ultimately, this will block the malignant progression of gastric cancer and improve the prognosis of patients with advanced gastric cancer.
